# RANKL Gene Polymorphism rs9594738 in Cases of Malocclusion Due to Persistence of Primary Teeth in Minangkabau Children

**DOI:** 10.1055/s-0045-1810422

**Published:** 2025-08-18

**Authors:** Fuccy Utamy Syafitri, Amin Akbar, Nila Kasuma, Reno Wiska Wulandari, Dwinda Rahmadhani

**Affiliations:** 1 Department of Orthodontics, Faculty of Dentistry, Andalas University, Padang, Indonesia; 2 Department of Psychology, Faculty of Psychology and Health, State University of Padang, Padang, Indonesia; 3 Department of Oral Biology, Faculty of Dentistry, Andalas University, Padang, Indonesia; 4 Department of Nutrition, Professional Dietisien Study Program, Semarang Ministry of Health Polytechnic, Padang, Indonesia

**Keywords:** permanent dental malocclusion, primary tooth persistence, RANKL gene polymorphism rs9594738, primary tooth resorption, DNA sequencing and electrophoresis

## Abstract

**Objective:**

Malocclusion is an important dental health problem, especially in children. One factor causing malocclusion is the persistence of primary teeth, which genetic factors can influence. This study provides a new understanding of the role of genetics in causing malocclusion and its impact on preventive planning and orthodontic treatment.

**Materials and Methods:**

This was an observational analytic study with a cross-sectional research design. The research subjects were children of SD Pembangunan UNP Padang, Minangkabau tribe, aged 6 to 13 years, a total of 30 people, consisting of a case group and a control group. Saliva was collected using a nonstimulated method (passive salivation). The polymorphism of the RANKL rs9594738 gene was analyzed using the polymerase chain reaction-restriction fragment length polymorphism method. Amplification results were analyzed via agarose gel electrophoresis to determine genotype. Data analysis was performed using the chi-square test.

**Results:**

RANKL rs9594738 gene polymorphism in the case group was higher than in the control group. The chi-square test shows an association between RANKL rs9594738 gene polymorphism and dental malocclusion due to the persistence of primary teeth.

**Conclusion:**

The data shows that the RANKL rs9594738 gene polymorphism is associated with dental malocclusion due to the persistence of primary teeth. The occurrence of malocclusion due to the persistence of primary teeth is a multigenetic phenomenon. In addition to the RANKL gene, osteoprotegerin, and matrix metalloproteinases, other genes that affect the replacement of primary teeth to permanent teeth are colony-stimulating factor 1, tumor necrosis factor ligand superfamily member 11, runt-related transcription factor 2, interleukin-1β, cathepsin K, sclerostin, and parathyroid hormone.

## Introduction

Malocclusion is a tooth or jaw position abnormality that is common in children. It can affect a person's masticatory function, facial appearance, and quality of life. One of the main causes of malocclusion is primary tooth persistence, a condition in which primary teeth do not fall out in time, hindering the growth of permanent teeth. 1

Genetic factors play an important role in causing malocclusion. One gene that has attracted attention is the receptor activator of nuclear factor-kappa B ligand (RANKL). The RANKL rs9594738 gene polymorphism has been associated with various bone-related conditions, including tooth and jaw development. 2 This study aims to determine the relationship between RANKL rs9594738 gene polymorphism and malocclusion caused by persistence of primary teeth in Minangkabau children.

## Primary Tooth Persistence and Malocclusion

Primary tooth persistence occurs when primary teeth do not fall out on time, even though the permanent teeth have erupted. This can be caused by the failure of the root resorption process of the primary teeth, which can be caused by genetic factors, ankylosis, or the absence of permanent tooth seeds. 1 Clinically, delayed eruption of permanent teeth is categorized as pathological if it exceeds the normal turnover time by more than 6 months. 3

Persistence of primary teeth can inhibit the eruption of permanent teeth, leading to malocclusions such as crowding, crowding, or overbite. Environmental, nutritional, and genetic factors may play a role in primary tooth persistence. 3

## The RANKL Gene and Its Function

The RANKL gene plays a role in bone metabolism by regulating osteoclast activity. Its expression is also associated with alveolar bone resorption, which is crucial for tooth replacement. Polymorphisms in the RANKL gene, especially rs9594738, have been reported to affect the risk of various bone and dental disorders. 2

## Minangkabau Tribe: Genetic Characteristics

The Minangkabau, or Minang, tribe is one of the ethnic groups of the archipelago located in the West Sumatra region. Traditional Minangkabau food is spiced and rich in texture. The eating habits of the people are influenced by geographical location, topographical conditions, traditions, and local beliefs. Minangkabau people are culturally agrarian rice farmers. Generally, the diet of an agrarian society consists of three meals a day, most of which come from crops and livestock. The Minangkabau people have distinctive genetic characteristics due to their history of migration and endogamous marriage practices. Genetic research in this population is important to identify genetic risk factors that affect their health. 4

## Materials and Methods

The sample is part of the population that meets the inclusion and exclusion criteria. The inclusion criteria were that the research subjects were native Minangkabau tribes from both parents (without mixing tribes), and were willing to become research subjects as evidenced by the signing of the willingness form by the patient or the patient's parents. Exclusion criteria included: patients using orthodontic devices, patients with agenesis, odontoma, and supernumerary teeth, patients with a history of congenital abnormalities, trauma or infection to the face, metabolic diseases, consumption of illegal drugs, patients with syndromic disorders, as well as research specimens that were damaged and could not be assessed.

This study was an observational analytic study with a cross-sectional design. The subjects were children of UNP Development Elementary School in Padang City, West Sumatra, Indonesia, with Minangkabau ethnicity and age range of 6 to 13 years. The minimum sample size was determined based on the two mean *t* -test formula, so the minimum sample size for each group was 30 subjects. The sample consisted of 15 children with malocclusion due to persistence of primary teeth (case group) and 15 children without malocclusion due to persistence of primary teeth (control group).

The control group was selected to match the age range of the case group, specifically corresponding to the period of permanent tooth eruption, in order to minimize potential confounding and improve the internal validity of the study. However, sex matching was not implemented due to limitations in sample availability. This limitation is acknowledged and discussed in the discussion section.

Saliva samples were collected using a nonstimulated passive drool method. Genotyping of the RANKL rs9594738 polymorphism was conducted using the polymerase chain reaction-restriction fragment length polymorphism method. The amplified products were analyzed by agarose gel electrophoresis to determine genotype profiles. Statistical analysis was carried out using the chi-square test.

## Results

### Characteristics of Research Subjects

**Fig. 1:**
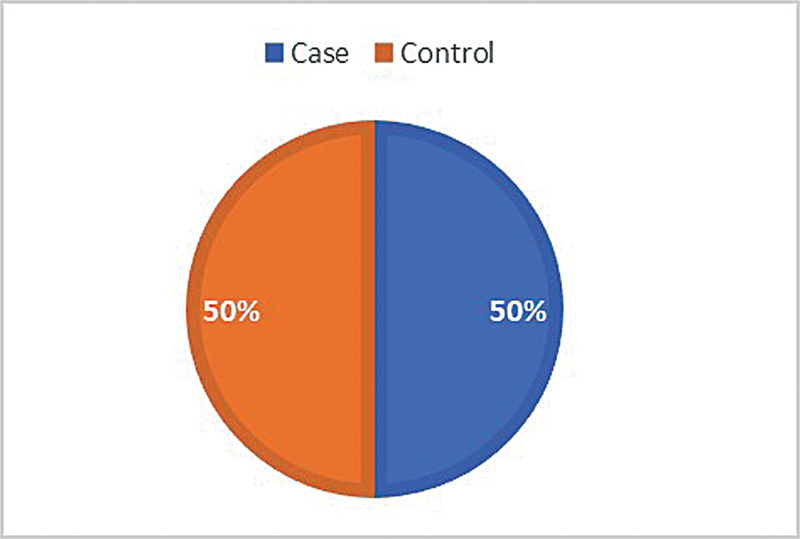
Overview of research subjects **.** The samples in this study were students of SD Pembangunan UNP in Padang City, a total of 30 people, who were divided into two groups: 15 subjects with malocclusion due to the persistence of primary teeth, called the case group, and 15 people who did not have malocclusion due to the persistence of primary teeth, called the control group ( Fig. 1 ).

The samples in this study were 30 students of SD Pembangunan UNP in Padang City, consisting of 15 people with malocclusion due to persistence of primary teeth (case group) and 15 people without malocclusion due to persistence of primary teeth (control group). Each group consisted of a number of male and female subjects ( Fig. 1 ). Subjects in the study of case groups and control groups were classified by gender, namely, female case groups and male case groups, as well as female control groups and male control groups.

### Frequency Distribution of RANKL rs9594738 Gene Polymorphism

Table 1 shows that the heterozygous mutant (CT) genotype in the RANKL rs9594738 polymorphism was found more in the case group compared to the control group. In this study, no homozygous mutant genotype (TT) was found. The statistical test results showed a significant difference between the two groups of research subjects, with a *p* -value = 0.026 ( *p* < 0.05), indicating an association between CT genotype and the incidence of primary tooth persistence.

**Table 1:** Frequency distribution of RANKL rs9594738 gene polymorphism

RANKL gene polymorphism rs9594738	Case group		Control group		Total		*p*	OR (95% CI)
	*n*	%	*n*	%	*n*	%		
Heterozygote (CT)	8	26.6	1	3.33	9	15	0.026	10.545 (1.227–90.662)
Wild-type (CC)	22	73.3	29	96.67	51	85		
Total	30	100	30	100	60	100		

Abbreviations: CI, confidence interval; OR, odds ratio.

Note: In Table 1 , it can be seen that in the case group there are more heterozygous mutant polymorphisms (CT) than the control group. In this study no homozygous mutant polymorphism was found. The test results showed a significant difference between the two groups of research subjects with a value of *p* = 0.026 ( *p* < 0.05).

### Differences in Mean Salivary RANKL Concentration Based on RANKL rs9594738 Gene Polymorphism

Table 2 shows that the mean salivary RANKL concentration in the group of subjects with heterozygous mutant polymorphism (CT) is higher than the group with wild genotype (CC). Statistical test results showed a significant difference between the two groups, with a value of *p* = 0.043 ( *p* < 0.05).

**Table 2:** Differences in mean salivary RANKL concentration based on RANKL rs9594738 gene polymorphism

RANKL gene polymorphism rs9594738	*n*	Average	± SD	*p*
CT (heterozygote)	9	549.52	0.163	0.043
CC (wild-type *)*	51	434.17	0.237	
Total	60			

Abbreviation: SD, standard deviation.

Note: Table 2 shows that the mean value of salivary RANKL concentration in the group of research subjects who experienced heterozygous mutant polymorphism (CT) was higher than that of wild-type research subjects (CC). The test results showed a significant difference between the two groups of research subjects with a value of *p* = 0.043 ( *p* < 0.05).

## Discussion

The results of this study indicate that the RANKL rs9594738 gene polymorphism plays a role in the persistence of primary teeth and contributes to the occurrence of malocclusion. The heterozygous genotype (CT) is thought to affect the root resorption mechanism of primary teeth, thus inhibiting the eruption of permanent teeth that should replace them. This finding is consistent with previous studies that have linked RANKL gene expression and variation to the process of bone resorption, including alveolar bone. 5

In addition to genetic factors, environmental influences such as diet, nutritional status, and oral habits are also known to play a role in the physiological process of tooth eruption. The interaction between genetic polymorphisms and environmental factors creates a complex dynamic that may increase an individual's susceptibility to primary tooth persistence and orthodontic complications, such as malocclusion. 6

In particular, the RANKL gene has a key role in regulating osteoclast differentiation and activity through the RANKL-RANK-osteoprotegerin (OPG) signaling pathway, which is critical in the alveolar bone remodeling process. Root resorption of primary teeth is a crucial step in paving the way for the eruption of permanent teeth. 6 Disruption of this pathway due to genetic variants such as rs9594738 may cause the roots of primary teeth to not degrade optimally, ultimately affecting the arrangement of permanent teeth. 7

These findings reinforce the importance of an integrative approach that considers both genetic and environmental factors in understanding the etiology of malocclusion, particularly in the Minangkabau population, which has its own genetic and cultural distinctiveness. 8

## RANKL and Osteoclast Activity

RANKL is a transmembrane protein that plays a crucial role in osteoclast differentiation, activation, and survival. The interaction of RANKL with the RANK receptor on osteoclast precursors initiates a signaling pathway that triggers the formation of active osteoclasts. This activation results in the release of enzymes such as cathepsin K (CTSK) that function to degrade the bone matrix. An imbalance in RANKL expression can disrupt osteoclast activity, which contributes to pathological abnormalities such as root persistence of primary teeth due to reduced bone resorption. 9

## Biological Mechanism of RANKL rs9594738 Polymorphism

The RANKL gene belongs to the tumor necrosis factor family that plays an important role in osteoclast differentiation, activation, and survival. In the process of tooth replacement, osteoclasts play a crucial role in the resorption of the roots of primary teeth to enable the eruption of permanent teeth. 10

Polymorphisms at the rs9594738 RANKL locus, located at specific chromosomal positions, may affect the expression level of the gene. The CT genotype of this polymorphism has been associated with delayed eruption of permanent teeth, which is thought to be due to decreased expression of RANKL as well as the RANKL/OPG ratio in the periapical tissues of permanent teeth. 11

## Effects of rs9594738 Polymorphism in RANKL

The rs9594738 polymorphism in the RANKL gene is an SNP variant (single-nucleotide polymorphism) that involves the replacement of one nucleotide at a specific position in the gene. This variant results in a CT genotype that can affect RANKL expression, thereby impacting osteoclast function. These changes in RANKL expression could potentially alter osteoclast activity in the bone resorption process, contributing to phenomena such as delayed eruption of permanent teeth or persistence of primary tooth roots. 10

## Effects of CT Genotype on Osteoclast Activity

The rs9594738 polymorphism with the CT genotype results in moderate levels of RANKL expression. The impact of the CT genotype on osteoclast activity can be explained through three main mechanisms 9 :

1. Moderate RANKL expression
The CT genotype produces sufficient levels of RANKL to support osteoclast activity, but tends to be lower than the CC genotype. This leads to a moderate effect on the bone resorption process. 12
2. Osteoclast differentiation
Differentiation of osteoclast precursors into active osteoclasts proceeds at a moderate rate, as the amount of RANKL available is sufficient to activate the RANK-nuclear factor kappa-B (NF-κB) signaling pathway, although it does not reach optimal levels. 13
3. Osteoclast activation and survival
  Activation of osteoclasts by RANKL in the CT genotype supports root resorption of primary teeth with sufficient efficiency. However, osteoclast survival is likely to be shorter than in the CC genotype, as the limited amount of RANKL means that osteoclast activity is not maximally maintained. Lower RANKL expression may also accelerate osteoclast apoptosis, thus reducing the number of active osteoclasts.

## Clinical Consequences of the RANKL rs9594738 Gene Polymorphism

1. Risk of primary tooth persistence
Decreased osteoclast activity in individuals with CT genotype increases the risk of primary tooth persistence compared to CC genotype. This condition inhibits the eruption process of permanent teeth, so that the primary teeth remain longer and potentially cause malocclusion. 10
2. Response to bone remodeling
Delayed alveolar bone remodeling in the CT genotype may worsen the condition of dental occlusion, which may require additional orthodontic intervention. However, the CT genotype is within the normal range and is not at risk of significant bone loss. 11
3. Response to clinical interventions
  Children with the CT genotype tend to show slower response to therapies aimed at stimulating bone resorption, such as pharmacological use of RANKL agonists or orthodontic appliances that facilitate bone remodeling.

## Effect of Polymorphisms on Osteoclast Function

The rs9594738 polymorphism in the RANKL gene affects osteoclast function by altering the regulation of RANKL expression, which impacts the following major biological pathways.

1. Osteoclast differentiation
  The decreased expression of RANKL due to this polymorphism inhibits the differentiation of osteoclast precursors into active osteoclasts, which is a crucial process for alveolar bone resorption.
2. Osteoclast activation
  Disruption of the interaction between RANKL and RANK receptors on osteoclast precursors reduces the signaling signals required to activate osteoclasts. As a result, the ability of osteoclasts to produce enzymes such as CTSK that are responsible for absorbing bone matrix is reduced.

## How the RANKL Gene Works in the Substitution of Primary to Permanent Teeth

The RANKL gene plays a crucial role in the process of primary to permanent tooth loss by regulating root resorption of primary teeth. This process occurs through complex molecular interactions between RANKL, its receptor RANK, and OPG as a RANKL antagonist. The balance between RANKL and OPG determines osteoclast activity in the alveolar bone, which in turn controls the root resorption process of primary teeth, enabling the eruption of permanent teeth. 14

## Mechanism of Action of RANKL Gene in Primary to Permanent Tooth Replacement

1. RANKL gene expression
  The RANKL gene is expressed in various tissues, including bone and periodontal ligament. Cells around the roots of primary teeth, such as fibroblasts and osteoblasts, increase RANKL production in response to mechanical stress from the underlying erupting permanent teeth. This increased expression of RANKL stimulates the tooth replacement process.
2. Interaction of RANKL and RANK
  RANK is a receptor found on the surface of osteoclast precursors. When RANKL binds to RANK, intracellular signaling pathways via NF-κB are activated, triggering the differentiation of precursors into mature osteoclasts ready for resorption.
3. Osteoclast activity in root resorption
  Activated osteoclasts attach to the root surface of primary teeth and release enzymes such as CTSK and hydrochloric acid that dissolve the mineral and organic matrix of the root. This resorption process leads to the reduction of the root of the primary tooth so that the tooth becomes loose and easily lost, making room for the permanent tooth to erupt.
4. Regulation by osteoprotegerin
  OPG is an antagonistic molecule that binds to RANKL thereby inhibiting the RANKL–RANK interaction. OPG thus controls osteoclast activity to keep root resorption in balance. The balance between RANKL and OPG is important to ensure normal tooth turnover.
5. Role of RANKL in timely tooth replacement
  RANKL expression is spatiotemporally regulated so that root resorption occurs at the right time and location. Disruption of RANKL expression, for example, due to genetic polymorphisms such as rs9594738, can slow or inhibit root resorption so that primary teeth stay longer and interfere with the eruption of permanent teeth.
6. Impact of abnormal RANKL activity
  If RANKL is overexpressed, root resorption proceeds too quickly resulting in premature loss of primary teeth and less space for permanent teeth, risking malocclusion. Conversely, low RANKL expression leads to late root resorption, causing persistence of primary teeth and inhibiting eruption of permanent teeth.
7. Clinical implications
  1. Early genetic diagnosis
    The RANKL rs9594738 genotype has the potential to be a screening biomarker to identify children at high risk of primary tooth persistence. This early detection allows for faster and more targeted preventive interventions.
  2. Multidisciplinary approach
    Treatment of primary tooth persistence should involve a multidisciplinary team consisting of geneticists, pediatric dentists, and orthodontists. The combination of genetic analysis and clinical evaluation can improve the accuracy of diagnosis and effectiveness of treatment.

Genetic testing for RANKL polymorphisms can help identify children at high risk of primary tooth persistence. RANKL-based therapies, such as the use of RANKL agonists, may increase osteoclast activity in cases with low RANKL expression. Conversely, OPG agonists can be used to inhibit osteoclast activity in cases of excessive resorption. With an in-depth understanding of the role of RANKL, dentists and orthodontists can plan earlier interventions for children with tooth replacement disorders, thus preventing complications such as severe malocclusion.

Apart from the RANKL gene, several other genes also play an important role in regulating the processes of root resorption, alveolar bone remodeling, and tooth replacement. Here are some other genes that affect primary tooth persistence 15 16 :

A. Osteoprotegerin gene
  OPG is a protein that functions as an inhibitor of RANKL activity by binding directly to RANKL, thus preventing its interaction with RANK receptors on osteoclast precursors. Thus, OPG decreases osteoclast formation and activity. Overexpression of OPG can inhibit root resorption of primary teeth by suppressing osteoclast activity, so that the roots of primary teeth remain intact and inhibit the eruption of permanent teeth. Several SNP variants in the OPG gene have been associated with impaired osteoclast function, which impacts the bone and tooth root resorption process.
B. Matrix metalloproteinases gene
Matrix metalloproteinases (MMPs) are a group of proteolytic enzymes that play an important role in the degradation of extracellular matrix, including collagen in primary tooth roots. Several types of MMPs, such as MMP-1, MMP-2, and MMP-9, are highly involved in the root resorption process. In the changeover from primary to permanent teeth, MMPs help to remodel the tissues around the roots of primary teeth, thus facilitating the eruption of permanent teeth. 17
  Disruption or imbalance of MMP activity can inhibit the root resorption process, resulting in delayed or failed primary tooth replacement. Specific mechanisms of how MMPs affect primary tooth replacement include:
  1. Function of MMPs in primary tooth root resorption
    MMPs, particularly MMP-1, MMP-2, MMP-9, and MMP-13, play a role in breaking down collagen types I and II, which are the main components of tooth root matrix and alveolar bone. MMP activity is essential in the root resorption process, especially in the degradation of collagen in the dentin of primary tooth roots. In addition, MMP-9 and MMP-13 also contribute to alveolar bone remodeling by breaking down the bone matrix, thus creating sufficient space for the growth and eruption of permanent teeth.
  2. Impaired MMP activity and primary tooth persistence
    Factors such as genetic polymorphisms, environmental influences, or regulatory imbalances may lead to dysfunction of MMP activity, which impacts primary tooth loss. The following are the mechanisms of impaired MMP activity:
    a. Decreased MMP activity
      Decreased MMP activity, which can be caused by genetic mutations or negative regulation, results in collagen in the tooth root not being optimally degraded. This causes the roots of primary teeth to remain intact and difficult to fall out. In addition, suboptimal alveolar bone remodeling due to low MMP activity inhibits the formation of adequate space for permanent tooth eruption.
    b. Excessive MMP activity
      Excessive MMP activity can cause root resorption of primary teeth to occur earlier than it should. This early resorption leads to premature loss of primary teeth, potentially disrupting the eruption guidance of permanent teeth and increasing the risk of malocclusion.
    c. Imbalance of MMP and tissue inhibitors of metalloproteinases activity
      Tissue inhibitors of metalloproteinases (TIMPs) function to control MMP activity. An imbalance between MMPs and TIMPs, such as excess TIMPs, can suppress MMP activity, slowing down the degradation process of the tooth root matrix. This inhibits root resorption and contributes to the persistence of primary teeth.
  3. MMP genetic polymorphisms affecting tooth turnover
    Several polymorphisms in MMP genes, specifically MMP-1, MMP-9, and MMP-13, have been associated with impaired enzyme activity that plays an important role in the process of tooth root resorption. MMP-1 is the main collagenase that breaks down type I collagen in tooth roots. Polymorphisms that decrease the expression or activity of MMP-1 can reduce the efficiency of root collagen degradation, thereby inhibiting resorption and causing retention of primary teeth.
    MMP-9 functions as a gelatinase that breaks down denatured collagen and elastin in the root matrix. Genetic polymorphisms that alter MMP-9 activity have the potential to slow down the root resorption process, thereby facilitating the persistence of primary teeth.
    Meanwhile, MMP-13 has a major role in the degradation of type II collagen in root bone and dentin matrix. Overexpression of MMP-13 can lead to excessive root resorption, while low expression can slow resorption, both of which result in the disruption of normal tooth replacement.
  4. Environmental factors affecting MMP activity
    In addition to genetic factors, MMP activity is also affected by various environmental factors. Lack of essential nutrients such as vitamin C and zinc can decrease MMP activity, thereby inhibiting the collagen degradation process that is crucial in tooth root resorption. Exposure to toxic chemicals, such as lead and mercury, is also known to suppress MMP expression, potentially impairing root resorption. Conversely, inflammatory conditions can trigger an excessive increase in MMP activity, which risks causing uncontrolled root resorption and damaging surrounding tissues.
  5. Therapies that potentially affect MMP activity
    Various therapeutic approaches can be applied to modulate MMP activity and support the tooth replacement process. MMP inhibitors: Specific inhibitors can be used to control rapid root resorption. MMP stimulation: Nutritional supplementation such as vitamin C, vitamin D, and zinc can increase MMP activity, especially in children with inhibited root resorption. Enzyme therapy: Therapies that enhance proteolytic activity may help accelerate collagen degradation in the roots of primary teeth that are difficult to fall out.
C. Colony-stimulating factor 1 gene
The colony-stimulating factor 1 (CSF1) gene encodes a protein that plays an important role in the production and differentiation of osteoclast precursors into mature osteoclasts. Decreased expression of CSF1 may inhibit the formation of sufficient osteoclasts, resulting in impaired root resorption of primary teeth and a slowdown in tooth replacement. 18
D. Tumor necrosis factor ligand superfamily member 11 gene
The tumor necrosis factor ligand superfamily member 11 (TNFSF11) gene encodes the RANKL protein, which is a major component in the regulation of osteoclast activity. Genetic variations in TNFSF11 can affect the expression level and function of RANKL, thus modulating the efficiency of tooth root resorption. Polymorphisms in this gene may increase or decrease the risk of primary tooth persistence. 19
E. Runt-related transcription factor 2 gene
Runt-related transcription factor 2 (RUNX2) is a major transcription factor that regulates osteoblast differentiation and bone formation. In addition, RUNX2 plays a role in the regulation of RANKL and OPG gene expression. Several polymorphisms in the RUNX2 gene have been associated with impaired alveolar bone growth and remodeling, which may impact the tooth replacement process. 20
F. Interleukin-1 beta gene
Interleukin-1 beta (IL-1β) is a proinflammatory cytokine that increases osteoclast activity by stimulating RANKL expression. Low IL-1β activity leads to decreased osteoclast stimulation, thus slowing down the root resorption process of primary teeth. Genetic variations in IL-1β affect the expression level of this cytokine, which impacts the speed and efficiency of root resorption during tooth replacement. 21
  Clinical implications: Identification of IL-1β polymorphisms can help detect a patient's risk of developing inflammatory-related root resorption disorders, so that appropriate interventions can be provided, especially in children with primary tooth persistence.
G. Cathepsin K gene
The CTSK gene encodes the enzyme CTSK, which is produced by osteoclasts to degrade the bone matrix and collagen in tooth roots during the resorption process. Mutations or polymorphisms in CTSK can decrease the enzyme activity, which has a direct impact on inhibiting root resorption of primary teeth. These genetic variations may result in ineffective root resorption, prolonging primary tooth residence and increasing the risk of malocclusion. 22
  Clinical implications: CTSK testing can be used to predict root resorption efficiency in patients, supporting appropriate therapy planning, such as stimulation of osteoclast activity or management of root resorption disorders.
H. Sclerostin gene
Sclerostin (SOST) encodes a sclerostin protein that functions to inhibit bone formation by blocking the Wnt signaling pathway, which is important in alveolar bone remodeling. Overexpression of sclerostin can slow down the process of alveolar bone remodeling and tooth root resorption, thus inhibiting the transition of primary to permanent teeth. Genetic variations in SOST may affect sclerostin expression levels and regulation of bone remodeling. 23
  Clinical implications: Detection of SOST polymorphisms may help assess the risk of impaired bone remodeling in children, so that interventions that support normal tooth replacement can be anticipated.
I. Parathyroid hormone gene
The parathyroid hormone (PTH) gene encodes PTH, which plays an important role in regulating blood calcium levels and the balance of bone metabolism. PTH affects osteoclast activity indirectly by regulating the expression of RANKL and OPG on osteoblast cells and other bone cells. Increased PTH will stimulate RANKL expression and suppress OPG, thereby increasing osteoclast activity and bone resorption, including root resorption of primary teeth. Conversely, genetic disruption or variation in the PTH gene may alter tissue sensitivity to this hormone and cause an imbalance between osteoclast and osteoblast activity, potentially inhibiting the root resorption process and slowing tooth turnover. 24

Clinical implications: Genetic variations in PTH may be a risk factor in tooth turnover disorders associated with suboptimal bone metabolism. Genetic testing of PTH may help design therapeutic strategies that support proper bone balance and root resorption.

Despite the strengths of this study, several limitations should be acknowledged. One important limitation concerns the selection of the control group. In this study, the control group was age-matched with the case group, focusing on the period of active eruption of permanent teeth—a critical phase for evaluating primary tooth persistence. This strategy was intended to minimize age-related confounding and enhance internal validity.

However, due to limited sample availability, sex matching was not implemented. This may be significant, as differences in sex distribution can influence gene expression and craniofacial development, potentially affecting outcomes related to tooth eruption and occlusion. Future research with larger and more balanced samples is recommended to account for both age and sex, thereby strengthening the validity of genetic associations in dental development.

## Conclusion

Primary tooth persistence is the result of complex interactions between various genes that regulate osteoclast activity, alveolar bone remodeling, and tooth root resorption. In addition to the RANKL gene, genes such as OPG, MMP, CSF1, RUNX2, IL-1β, and CTSK also have important roles in the process. Polymorphisms in these genes can affect the molecular pathways involved, thereby increasing or decreasing the risk of primary tooth persistence.

The RANKL gene plays a crucial role in the transition from primary to permanent dentition by regulating osteoclast activity in the RANKL–RANK pathway. Balanced expression of RANKL is essential to ensure the root resorption process takes place in a timely manner, allowing normal eruption of permanent teeth. Disruption of RANKL expression or function, whether caused by genetic or environmental factors, can inhibit the process and potentially increase the risk of malocclusion. Therefore, further research into the mechanism of action of RANKL is urgently needed to develop innovative therapies to treat tooth replacement disorders.

MMP also plays a significant role in root resorption of primary teeth by breaking down collagen and extracellular matrix components. Imbalances in MMP activity, which can be caused by genetic factors, environmental influences, or regulation by inhibitors such as TIMP, can inhibit the process of changing from primary to permanent teeth. Deepening research related to the modulation of MMP activity is expected to open up opportunities for the development of more effective therapeutic solutions to overcome the persistence of primary teeth and the accompanying complications of malocclusion.
